# Evaluation of the Effectiveness of a Group CBT-Based Intervention Aiming to Reduce Self-Stigma and Improve Recovery-Related Outcomes in People with Severe Mental Disorders: Randomised Controlled Trial

**DOI:** 10.1007/s11126-024-10092-9

**Published:** 2024-10-08

**Authors:** Katarzyna Frączek-Cendrowska, Piotr Świtaj, Izabela Stefaniak

**Affiliations:** 1 First Department of Psychiatry, Institute of Psychiatry and Neurology, Warsaw, Poland; 2 Maria Sklodowska – Curie Medical Academy, Warsaw, Poland; 3 Faculty of Medicine, Lazarski University, Warsaw, Poland

**Keywords:** Internalised stigma, Anti-stigma intervention, CBT-based group intervention, Sense of coherence, Stigma resistance

## Abstract

Self-stigma is a major barrier to personal and clinical recovery in people with mental illness. Although psychosocial interventions have been developed to reduce self-stigma, the exploration of group CBT-based interventions for hospitalised patients are less represented. The purpose of this trial is to investigate the effectiveness of a group CBT-based intervention aiming to reduce self-stigma and improve recovery-related outcomes such as self-esteem, stigma resistance and sense of coherence, which comes down to saying, “I am what I am”. A total of 104 patients admitted to the inpatient therapy were recruited to participate in a randomised controlled clinical trial, and 77 participants (46 in the intervention group and 31 in the control group) completed the trial. Constructs of interest were measured before and after the intervention. The results showed that the evaluated intervention was effective in improving sense of coherence and stigma resistance, compared to treatment as usual (TAU). The level of self-stigma significantly decreased in both the intervention and control groups, but no statistically significant difference was observed between the groups. The limitations of the study include: the lack of assessment of the sustainability of the effects of therapy (follow-up), the presence of intensive interventions of the ward’s programme and patients pre-qualified for the ward based on admission criteria. The intervention “I am what I am,” has proven to be effective, especially in increasing the level of personal resources needed to build clinical and personal recovery and to counteract the negative consequences of self-stigma.

## Introduction

The diagnosis of a severe mental disorder is associated with a process of stigmatisation [1]. The effect of internalising stigma and accepting it is self-stigma (also referred to as internalised stigma) [2–7]. Available studies show how stigma, depending on its severity, impacts personal recovery in people with severe mental illnesses (SMI) which is described as a multidimensional process consisting of clinical, existential, functional, physical, and social aspects [8–10]. The social-cognitive model of self-stigma describes it as consisting of stereotypes, prejudice, and discrimination – much like public stigma [11]. People with mental illnesses have preconceptions about themselves and tend to agree with stereotypes about mental illness. Prejudice leads to negative emotional reactions, including low self-esteem and self-efficacy [12, 13]. Due to these preconceptions, people with mental health problems may be at risk of failing to achieve previously set life goals which, as a vicious circle, becomes a factor that reinforces a low sense of self-worth [14–17]. Reflecting on the described self-stigma mechanism makes it possible to see the key role of self-esteem as its level is one of the main factors in recovery [18].

The course of recovery is also considered in terms of models focusing on the patient’s personal resources and strengths [19]. Factors supporting recovery include a sense of self-worth, a sense of coherence, or stigma resistance. Research shows that interventions increasing personal resources have an impact on reducing internalised stigma [20]. The negative effects of self-stigmatisation among people undergoing psychiatric treatment include an exacerbation of symptoms, an increased sense of loneliness, and a reduction in personal resources such as self-esteem, self-efficacy and hope, and a decrease in quality of life [21–26]. As many as 42% of people with schizophrenia and around 22% of people with diagnosed affective disorder show moderate to high levels of self-stigma [27, 28]. Higher levels of internalised stigma are associated with patients adopting coping strategies to deal with their mental illness diagnosis by hiding it [29, 30]. Although the self-stigma associated with a diagnosis of severe mental illness is a major and increasingly recognised problem in clinical practice, we still have little influence on the factors that could measurably reduce this process. Therefore, educational and therapeutic interventions aimed at reducing self-stigma, strengthening personal resources, and increasing stigma resistance, thus supporting the healing process, are becoming important [20, 31]. In the present trial, we also focus on factors oriented towards promoting personal recovery. A sense of coherence is associated with Antonovsky’s salutogenetic and therefore health-oriented approach [32]. Antonovsky’s theory of the “sense of coherence (SOC)” is used to explain the apparent contradiction of people with severe illnesses reporting a subjective experience of good health. A high SOC indicates the ability to understand the world in a way that facilitates effective coping with countless and complex stressors we face throughout our lives [32]. Sense of coherence was also assessed in the population of people with mental disorders. Findings indicated SOC as a good predictor of quality of life in people treated for schizophrenia. It is regarded as a variable that promotes recovery in people with severe mental disorders [33, 34]. Another factor influencing recovery is stigma resistance, which is defined as the ongoing, active process of using one’s experiences, skills, and knowledge to develop a positive self-image [35, 36].

The effectiveness of interventions aimed at reducing self-stigma in people with mental illnesses is systematically reviewed. The interventions described in literature vary in their initial assumptions. Interventions against self-stigma vary in form (group [37–40], individual [41, 42], or a combination of the two [43]); duration (from one 3-h meeting [39] to 20 weekly meetings [40, 44, 45], or in the theoretical assumptions underlying the creation of the programme. Several interventions aimed at helping individuals reduce internalisation of stigmatising beliefs have been developed. Many include psychoeducation to correct negative beliefs about mental illness, cognitive techniques to combat self-stigmatising thoughts, reinforcement of personal strengths, and practical skills for coping with self-stigma [20]. Psychoeducational and multicomponent interventions have proved to be the most effective [37, 38, 43, 46, 47]. Studies of the effectiveness of cognitive-behavioural interventions [41, 42] and those oriented towards revealing the diagnosis [48, 49] have yielded inconclusive results. The most widely used psychoeducational interventions include psychoeducation about mental health and mental disorders, aspects and effects of stigma, its consequences and strategies for coping with stigma [20]. Multicomponent interventions combine elements of different models, e.g., psychoeducation, cognitive-behavioural therapy (CBT), motivational interviewing, and social skills training [43]. Recently, there has been an increase in the number of interventions that rely on the assumptions of “third-wave” therapeutic cognitive-behavioural therapy e.g. self-compassion or mindfulness [50]. According to a review of literature performed by Mittal et al. [51] there is a paucity of interventions targeting self-stigma that can be applied in a hospital setting, as well as CBT-based group interventions. This trial aims to evaluate the effectiveness of a CBT-based group intervention against self-stigma delivered in a psychiatric hospital setting. The main hypothesis was: compared to the control group, patients from the experimental group will show a lower level of self-stigma upon completing the intervention. The additional hypotheses were: upon completing the intervention, when compared to the control group, patients in the experimental group will demonstrate higher level of stigma resistance, lower tendency to hide mental illness, higher level of self-esteem, higher level of sense of coherence, lower level of loneliness, higher level of life satisfaction, lower overall severity of psychopathological symptoms.

## Material and Methods

### Inclusion Criteria

The intervention against self-stigma “I am what I am” (abbr.: the intervention) was conducted in inpatient wards with a psychotherapeutic profile at the Institute of Psychiatry and Neurology in Warsaw, Poland. It included patients hospitalised in wards meeting the inclusion criteria, who gave informed consent to participate in the trial. The trial included people with severe mental illness – psychotic and affective disorders (diagnosis codes F20 – F29 and F30 – F39 according to the International Classification of Diseases and Health Problems, ICD-10).

Other inclusion criteria included:

- age over 18,
- no co-existing active psychoactive substance dependence,
- no documented intellectual disability or organic impairment of the central nervous system,
- a stable mental state, that allows the use of the anti-stigma intervention (patients without acute psychiatric symptoms).

### Procedure

The trial was conducted between May 2021 and July 2022. The Bioethics Committee at the Institute of Psychiatry and Neurology granted consent for the trial (Resolution No. 33/2020 of 18.11.2020). Patients were eligible for admission to the ward based on an initial interview with an experienced psychiatrist, where the main criterion for admission was experiencing an affective and/or psychotic crisis.

Patients qualified for admission to the ward were informed about the possibility of participating in the "I am what I am" intervention, during individual meetings with a psychiatrist. The intervention was included as an additional component of the ward’s therapeutic programme including: therapeutic community meetings, psychoeducation, therapeutic groups, metacognitive training, initiative workshops, physical activity training, and cognitive training.

Patients received a written and oral description of the "I am what I am" intervention and the study. Then, after patients declared their willingness to participate in the "I am what I am" intervention, they were asked about consent to participate in the study. After obtaining informed consent, patients were assigned to one of two groups: experimental group (E) or control group (C).

The trial conducted was a randomised controlled trial with a blinded outcome assessment of the effect of the intervention and two measurement points of T0 (performed before the intervention) and T1 (performed 5 weeks later, post intervention). The primary endpoint indicator was level of internalised stigma. The secondary endpoint indicators were: stigma resistance, secrecy about one’s mental illness, loneliness, sense of coherence, level of life satisfaction, self-esteem, and psychopathological symptoms.

After assessment at examination point T0, subjects were randomly (www.randomizer.org) allocated to one of two groups:

A. Experimental group (E) – patients were immediately involved in a therapeutic intervention “I am what I am”, lasting 5 weeks (10 therapy sessions conducted twice a week), in addition to participating in other interventions offered during hospitalisation;
B. Control group (C) – after examination at T0, patients participated for 5 weeks in the programme offered during hospitalisation without self-stigma intervention.

At the end of the 5-week intervention or treatment as usual period, patients were examined at T1 (with the same scales as at point T0) by a clinician (psychiatrist), who did not know the allocation to the intervention or control group of the trial participants.

**Fig. 1:**
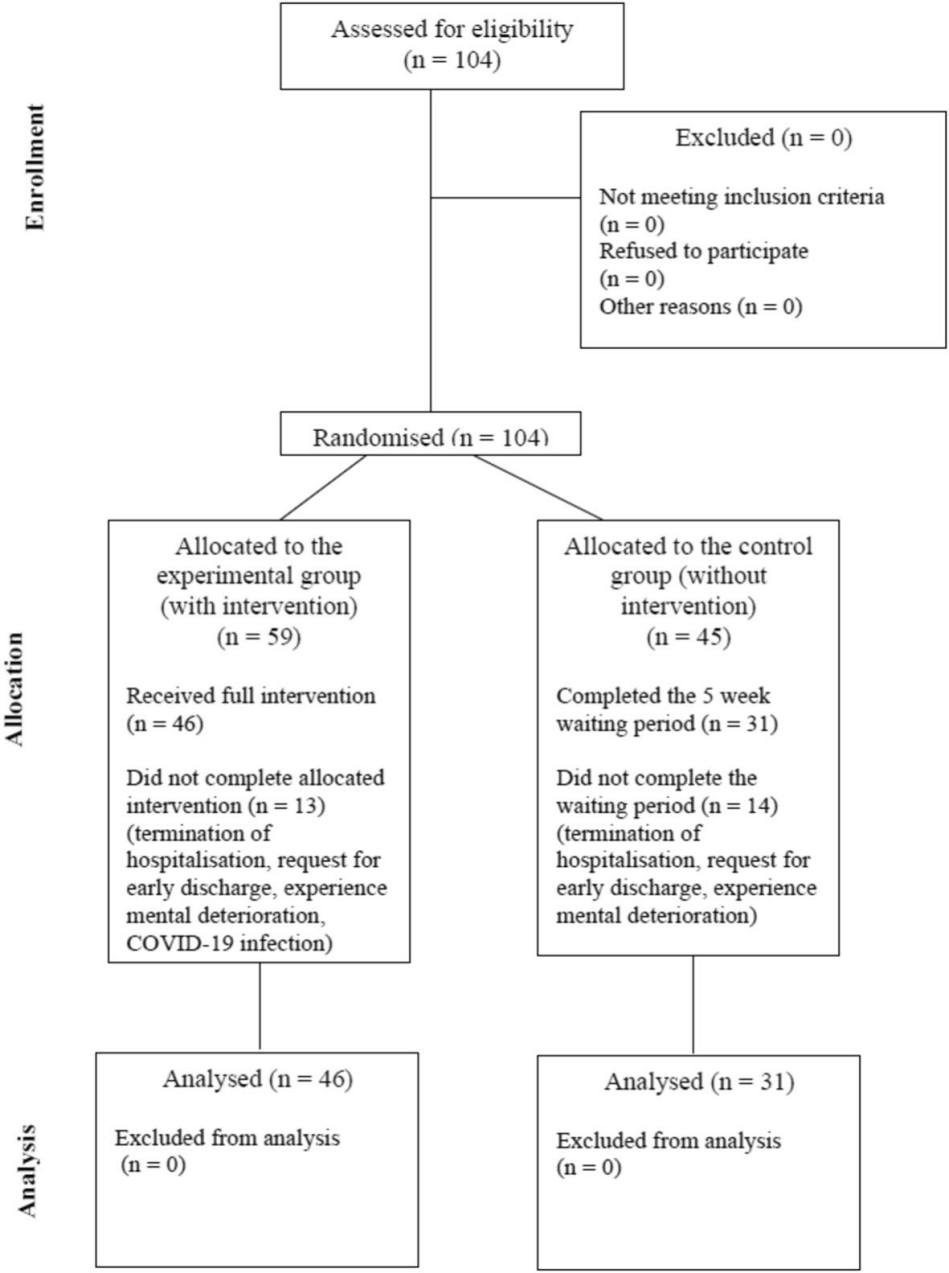
Diagram showing the course of the trial

The course of the trial is shown in Fig. 1.

### Description of “I Am What I Am” Intervention Against Self-Stigma

The intervention “I am what I am” took place in a group format, led by a therapist and a co-therapist. As part of the “I am what I am” intervention, patients attended 10 sessions of 60 min each, held twice a week, so that patients could benefit fully during their hospitalisation. Each session was conducted according to the protocol described in Appendix, incorporating a cognitive-behavioural approach, in which psychoeducation and a module on therapeutic work on improving self-esteem became important elements [44]. This protocol was previously presented in the article [52]. Due to formal considerations regarding the patients’ hospitalisation the trial conducted did not choose to adapt interventions already in place, such as *Narrative Enhancement and Cognitive Therapy* NECT [40, 53].

### Measures

The tools used in the trial included:

Self-report scales:

- Self-stigma was assessed using the Internalised Stigma of Mental Illness scale (ISMI) [54]. This questionnaire consists of 29 items, grouped into five sub-scales: alienation (example item: “I feel out of place in the world because I have a mental illness”), stereotype endorsement (example item: “Mentally ill people tend to be violent”), discrimination experience (example item: “People discriminate against me because I have a mental illness”), social withdrawal (example item: “I avoid getting close to people who don’t have a mental illness to avoid rejection”) and stigma resistance (example item: “I can have a good, fulfilling life despite my mental illness”). Answers to the ISMI statements are given on a four-point scale ranging from 1 (definitely disagree) to 4 (definitely agree). In the present study, Cronbach’s alpha for the ISMI total score was found to be 0.93.
- Stigma resistance was measured by means of the Stigma Resistance Scale (SRS) [55]. The SRS is a 20-item questionnaire containing five subscales: self-other differentiation (example item: ‘I can have a positive view of myself even when others don’t have a positive view of me’), personal identity (example item: ‘My diagnosis does not define me’), personal cognitions (example item: ‘I challenge negative thoughts that I may have about myself related to having a mental illness’), peer stigma resistance (example item: ‘I help others see they should not be ashamed about mental illness’), and public stigma resistance (example item: ‘I advocate for better treatment for people with mental illness’). Participants mark their responses on a scale from 1 (disagree) to 5 (agree). Only the overall SRS score was used in the current analyses (Cronbach’s alpha = 0.90).
- The extent to which participants endorse concealment as a means of avoiding rejection because of having mental illness or receiving psychiatric treatment was evaluated with the Secrecy Scale (SS) [56]. This instrument is composed of nine items to which responses are given on a four-point scale (1 = strongly agree; 4 = strongly disagree). Example item is: “In view of society’s negative attitudes towards people with serious mental illnesses, you would advise people with serious mental illnesses to keep them a secret“. In our data, Cronbach’s alpha for this scale was 0.88.
- Self-esteem was assessed using the Rosenberg Self-Esteem Scale (RSES) [57]. This is a 10-item questionnaire with a four-point response scale (1 = strongly agree; 4 = strongly disagree). Example item is: “I feel that I have a number of good qualities”. The RSES demonstrated good internal consistency in our sample (Cronbach’s alpha = 0.83).
- Sense of coherence (SOC) was measured by means of the 29-item Sense of Coherence Scale (SOC-29) [32]. This tool comprises of three components: comprehensibility, manageability and meaningfulness. Respondents are asked to select a response on a seven-point semantic differential scale with two anchoring phrases. Example item assessing comprehensibility is: “Do you have a feeling that you are in an unfamiliar situation and don’t know what to do? (from ‘very often’ to ‘very seldom or never’)”. Manageability is measured with items like: “When something unpleasant happened in the past your tendency was: (from ‘to eat yourself up about it’ to ‘to say “ok that’s that, I have to live with it” and go on’)”. The following item is an example for meaningfulness component: “When you think about your life, you very often: (from ‘feel how good it is to be alive’ to ‘ask yourself why you exist at all’)”. Per the recommendations of Antonovsky, who considered the SOC-29 a measure of global orientation to life, only a total scale score was utilised in the analyses. In the current sample, the value of Cronbach’s alpha for the SOC-29 was 0.91.
- Loneliness was assessed by the De Jong Gierveld Loneliness Scale (DJGLS) [58]. This measure consists of 11 items, to which interviewees are asked to respond on a five-point scale ranging from 1 (yes!) to 5 (no!). Example item is: “I miss having people around me”. It can be used to assess both the overall level of loneliness and two of its dimensions: emotional and social. In this study, only the total scale score was included in the analyses (Cronbach’s alpha = 0.89).
- Satisfaction with one’s life was examined using the Satisfaction with Life Scale (SWLS) [59]. This is a short five-item questionnaire answered on a seven-point scale (1 – strongly disagree; 7 – strongly agree). Example item is: “In most ways my life is close to my ideal”. In our study, Cronbach alpha coefficient for the SWLS was 0.83.

Clinical assessment:

- The overall severity of psychopathological symptoms was measured with the standard version of the Brief Psychiatric Rating Scale (BPRS) [60]. It consists of 18 items rated by a clinician on a scale from 1 (symptom not present) to 7 (symptom extremely severe). Cronbach’s alpha for the BPRS was found to be 0.71.

For all instruments, higher total scores indicate higher levels of the measured constructs.

The same tools were used at examination point T0 and T1.

## Results

### Data Analysis

The statistical analysis was carried out in three steps. In the first, calculations included descriptive statistics, such as means, standard deviations (SD), and Pearson correlations. Univariate analyses were carried out using the Student’s t-test and Wilcoxon test for continuous variables according to their distributions. Nominal variables were analysed by contingency tables using the Chi-squared test or Fisher’s exact test.

In the next step, single imputation methods were used to replace missing data when the proportion of missing data was insignificant (according to ICH E9 guidelines – Statistical Principles of Clinical Trials). As an imputation model, the Approximate Bayesian Bootstrap (ABB) technique was used incorporating possible parameter bias into the imputation hot deck model. Mixed models in which explicit imputation is not used (EMA Guideline on Missing Data in Confirmatory Clinical Trials) were used in the absence of repeated measurement data.

In the third step, linear mixed models for repeated measures with a compound symmetry structure of the correlation matrix were used to test for differences between groups. The models tested for differences between factor levels using appropriate contrasts.

The power analysis was performed using following approach [61]: (a) a fixed alpha level (*p* = 0.05), (b) the sample size planned in the study (n = 100), (c) a "meaningful" unstandardised mean difference effect, and (d) an estimate of the error variance obtained from data of the preliminary pilot study. For the SRS scale, with an expected effect size between medium and large (η2 = 0.12), a power of 90% was obtained. Similarly, for the SOC scale, for which the expected effect size is in the medium to large range (η2 = 0.13), a power of 90% was obtained. For the ISMI scale, for an effect size between small and medium (η2 = 0.04), a power of 40% was obtained.

The calculations were performed in the SAS suite (Analytical Products rel. 15.1). A value of *p* < 0.05 was adopted as a significance level.

### Demographic and Clinical Data of the Group

The trial involved 104 participants, with 59 participants in the experimental group (E) and 45 participants in the control group (C) completing the trial, and 27 participants not completing the trial (13 participants from the experimental group and 14 participants from the control group). In the experimental group, the mean age of participants was 35.2 years (SD = 11.2) and in the control group 35.1 years (SD = 12.3). The groups did not differ significantly in terms of age.

Data on sex, living situation, education, place of residence, or work situation are shown in Table 1. The groups were not statistically significantly different in terms of sociodemographic variables, the exception being place of residence (*p* = 0.026).

**Table 1:** Sociodemographic characteristics of subjects

	Experimental group (E)		Control group (C)		Statistical significance (*p*-value)
	***N***	***%***	***N***	***%***	
Sex					
Women	29	49%	20	44.4%	0.694
Men	30	51%	25	55.6%	
Marital status					
Single	46	78%	34	75.6%	0.949
Marriage	6	10.2%	4	8.9%	
Unmarried partnership	4	6.8%	3	6.7%	
Divorce	3	5.1%	3	6.7%	
Separation	0	0%	1	2.2%	
Widowhood	0	0%	0	0%	
Education					
Higher	28	47.5%	17	37.8%	0.427
Secondary education	27	45.8%	21	46.7%	
Vocational	2	3.4%	5	11.1%	
Primary	2	3.4%	2	4.4%	
Employment status					
Employment	10	17%	5	11.1%	0.543
Unemployment	14	23.7%	12	26.7%	
Student	5	8.5%	2	4.4%	
Retirement pension	2	3.4%	2	4.4%	
Social security pension	24	40.7%	22	48.9%	
Other	3	3.9%	1	2.0%	
Place of residence					
Village	12	20.3%	11	24.4%	0.026
City of up to 50,000 inhabitants	17	28.8%	10	22.2%	
City of 50–100 thousand inhabitants	1	1.7%	8	17.8%	
City of 100–500 thousand inhabitants	2	3.4%	3	6.7%	
City of > 500 thousand inhabitants	27	45.8%	13	28.9%	

*N* total number, *p* statistical significance

The trial involved patients with diagnoses from F20.0–F29.0 and F30.0–F39.0. The distribution of diagnoses in each group is given in Table 2. The mean duration of psychiatric treatment in group (E) was (M = 12.3); in group (C) – (M = 11.8). The mean age of beginning treatment in group (E) was 22.9 years (SD = 6.8); while in group (C), it was 23.7 years (SD = 10.8). The groups were not statistically significantly different in the clinical parameters described. The results are presented in Table 2.

**Table 2:** Clinical data of trial participants

Factor	Experimental group (E)		Control group (C)		Statistical significance (*p*-value)
Diagnosis	*N*	%	*N*	%	
F20.0–F29.0	50	84.8%	41	91.1%	0.384
F30.0–F39.0	9	15.2%	4	8.9%	
	**M**	**SD**	**M**	**SD**	
Start of treatment – age	22.9	6.8	23.7	10.8	0.555
Treatment time – years	12.3	10.2	11.8	8.6	0.876

*N*  total number, *M*  arithmetic mean, *SD*  standard deviation, *p*  statistical significance

The trial reported the reasons for not completing the trial, i.e., 11 people terminated their hospitalisation, 11 people requested early discharge, 4 people experienced mental deterioration (these were 3 people in the control group and 1 person in the experimental group), and one person discontinued the trial due to COVID-19 infection.

The mean scores obtained on the scales used in the trial in both the experimental and control groups at measurement points T0 and T1 are shown below *(*Table 3).

**Table 3:** Mean scores and standard deviations in the experimental and control groups at the two measurement points

Scale (overall result)	Group (E, C)	T0		T1	
		*N*	M (SD)	*N*	M (SD)
ISMI	E	55	2.3 (0.4)	46	2.2 (0.3)
	C	43	2.4 (0.5)	31	2.3 (0.4)
SRS	E	57	71.3 (14.4)	44	78.1 (11.7)
	C	43	72.3 (12.4)	31	71.6 (11.4)
SS	E	58	23.2 (4.9)	45	22.2 (5.9)
	C	45	23.3 (5.3)	31	22.3 (4.7)
RSES	E	58	25.0 (4.2)	46	26.7 (3.5)
	C	43	24.6 (4.9)	31	25.3 (3.4)
SOC-29	E	56	111.4 (25.8)	46	120.7 (23.2)
	C	42	111.8 (27.1)	30	111.1 (24.2)
DJGLS	E	59	32.7 (8.7)	46	31.2 (7.5)
	C	45	32.7 (8.4)	31	32.3 (8.2)
SWLS	E	58	16.4 (6.4)	45	18.3 (5.8)
	C	43	16.7 (6.6)	31	16.6 (6.4)
BPRS	E	59	33.0 (8.3)	45	27.2 (8.1)
	C	44	36.1 (8.5)	31	29.2 (7.5)

*N* total number, *M* arithmetic mean, *SD* standard deviation, *T0 *first examination point, *T1 *second examination point, *ISMI *Internalised Stigma of Mental Illness scale, *SRS *Stigma Resistance Scale, *SS *Secrecy Scale, *RSES *Rosenberg Self-Esteem Scale, *SOC-29 *The Sense of Coherence Questionnaire, *DJGLS *De Jong Gierveld Loneliness Scale, *SWLS *Satisfaction with Life Scale, *BPRS *Brief Psychiatric Rating Scale

### Analysis of Mixed Linear Models for the Measurement Scales Used

#### Primary Endpoint

The Internalised Stigma of Mental Illness scale (ISMI) showed decreases in scores (reduction in self-stigma) in the experimental and control groups between measures T0 and T1. In the experimental group, the change in value was -0.15 ± 0.04; while in the control group, it was -0.10 ± 0.05. These changes were statistically significant, with *p-*values of 0.0003 and 0.0360, respectively.

The two groups were not statistically significantly different at T0 and T1, the differences being 0.10 ± 0.08 and 0.15 ± 0.09, respectively.

**Fig. 2:**
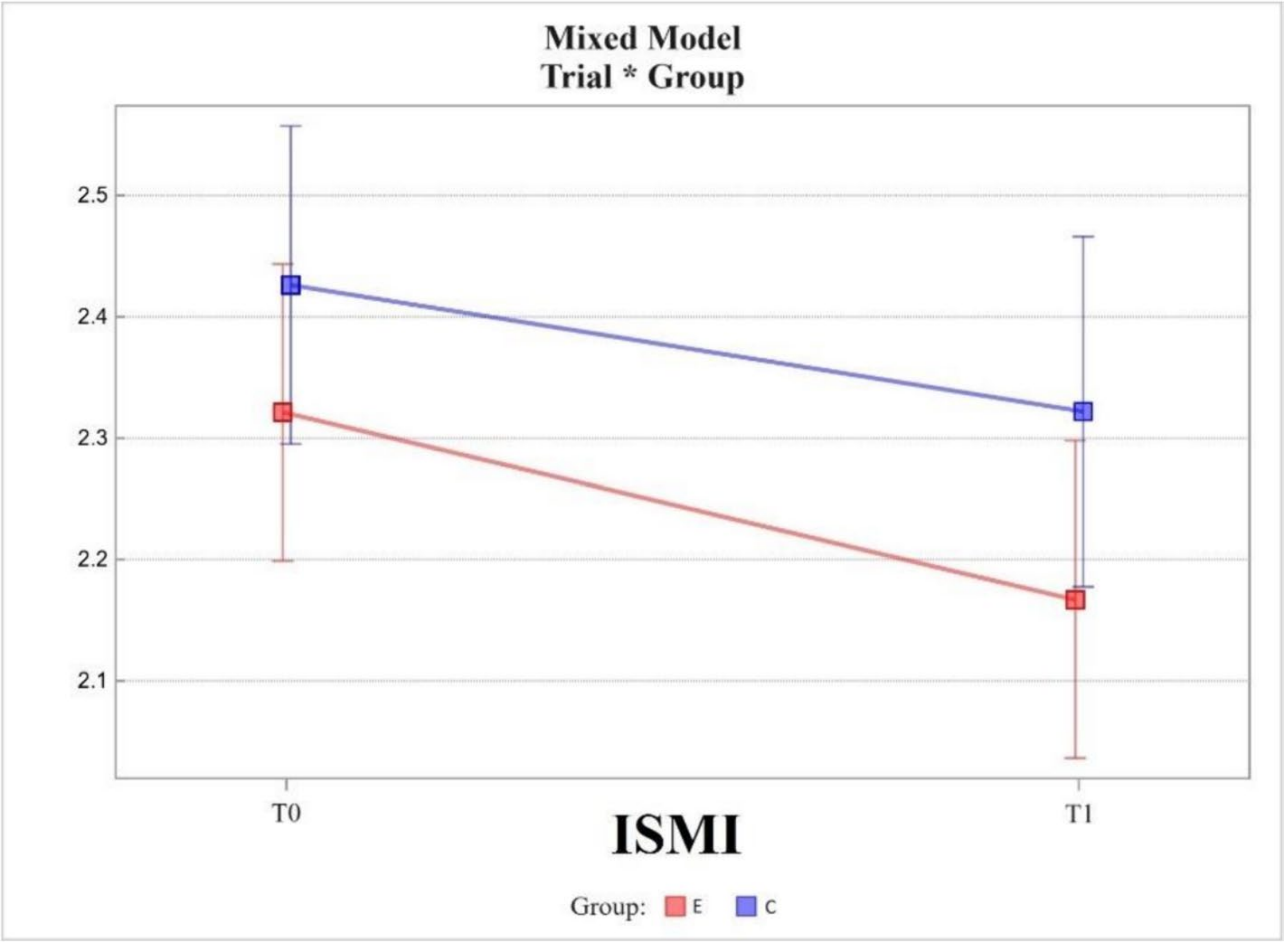
Mixed model – Internalised Stigma of Mental Illness scale (ISMI); measurement points T0 and T1

The change in scores between T0 and T1 measurements in the experimental group did not differ from that in the control group, the difference between the two being -0.05 ± 0.06 *(*Fig. 2).

#### Secondary Endpoints

The Rosenberg Self-Esteem Scale (RSES) range showed increases in scores (increases in self-esteem) in the experimental and control groups between measures T0 and T1. In the experimental group, the change in value was 1.64 ± 0.45; while in the control group, it was 1.01 ± 0.55. The change was statistically significant only in the experimental group (*p* < 0001).

The difference between groups at T0 was 0.32 ± 0.84 and at T1 0.95 ± 0.90. Both differences were not statistically significant.

**Fig. 3:**
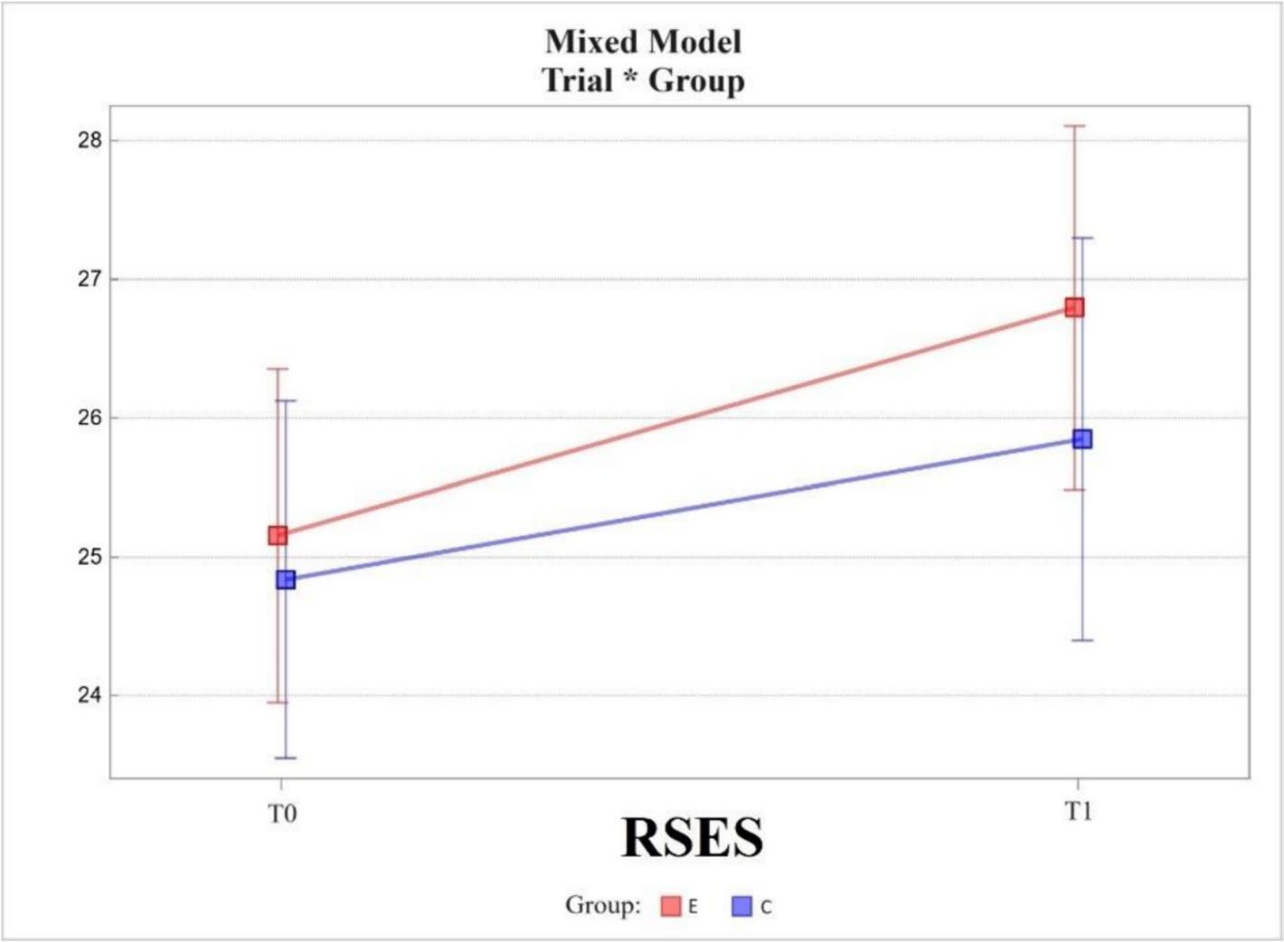
Mixed model – Rosenberg Self-Esteem Scale (RSES); measurement points T0 and T1

The change in scores between T0 and T1 measurements in the experimental group did not differ from that in the control group, the difference between the two being 0.63 ± 0.70 *(*Fig. 3).

The Sense of Coherence Questionnaire (SOC-29) showed an increase in scores (increase in sense of coherence) in the experimental group and a decrease (decrease in sense of coherence) in the control group between measurements at T0 and T1. In the experimental group, the change in value was 8.29 ± 2.32; while in the control group, it was -0.60 ± 2.82. The change was statistically significant only in the experimental group (*p* = 0.0006).

The groups were not statistically significantly different in the area tested with the SOC Questionnaire at T0, the difference being 2.07 ± 5.11. The difference in scores between the groups at T1 was 6.82 ± 5.42 and was not statistically significant.

**Fig. 4:**
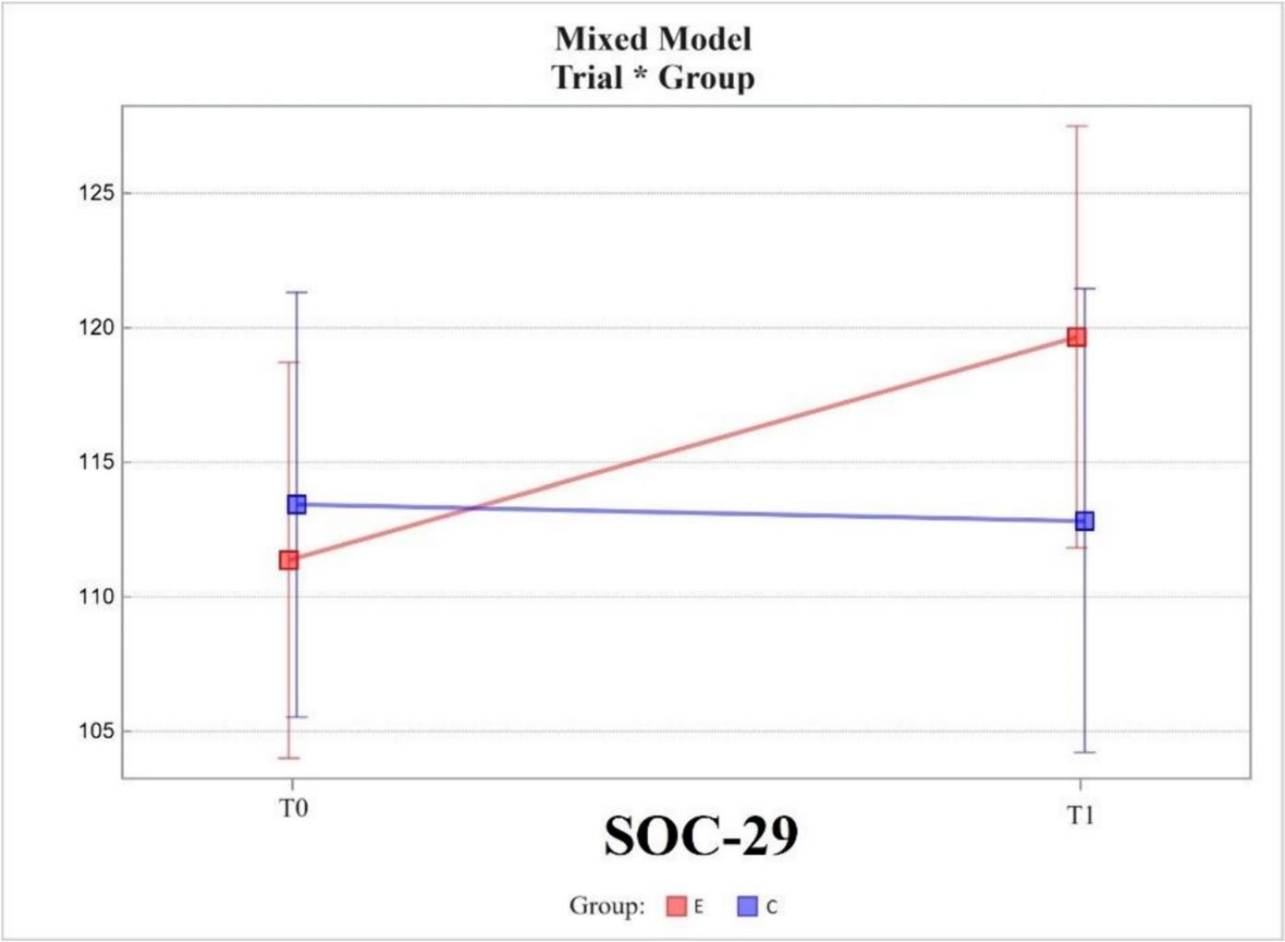
Mixed model – The Sense of Coherence Questionnaire (SOC-29); measurement points T0, T1

The change in scores between the T0 and T1 measurements in the experimental group differed from that in the control group, the difference between the two being 8.89 ± 3.63, which was statistically significant; *p* was 0.0166 (Fig. 4).

The Stigma Resistance Scale (SRS) between measurements T0 and T1 showed an increase in scores (increase in stigma resistance) in the experimental group and a decrease (decrease in stigma resistance) in the control group. The change in value was 5.99 ± 1.62 in the experimental group and -2.01 ± 1.96 in the control group. The change was statistically significant only in the experimental group (*p* = 0.0004).

The groups were not statistically significantly different in the area tested with the SRS scale at T0, the difference being 2.11 ± 2.51. The difference in scores between groups at T1 was 5.89 ± 2.8 and was statistically significant (*p* = 0.0356).

**Fig. 5:**
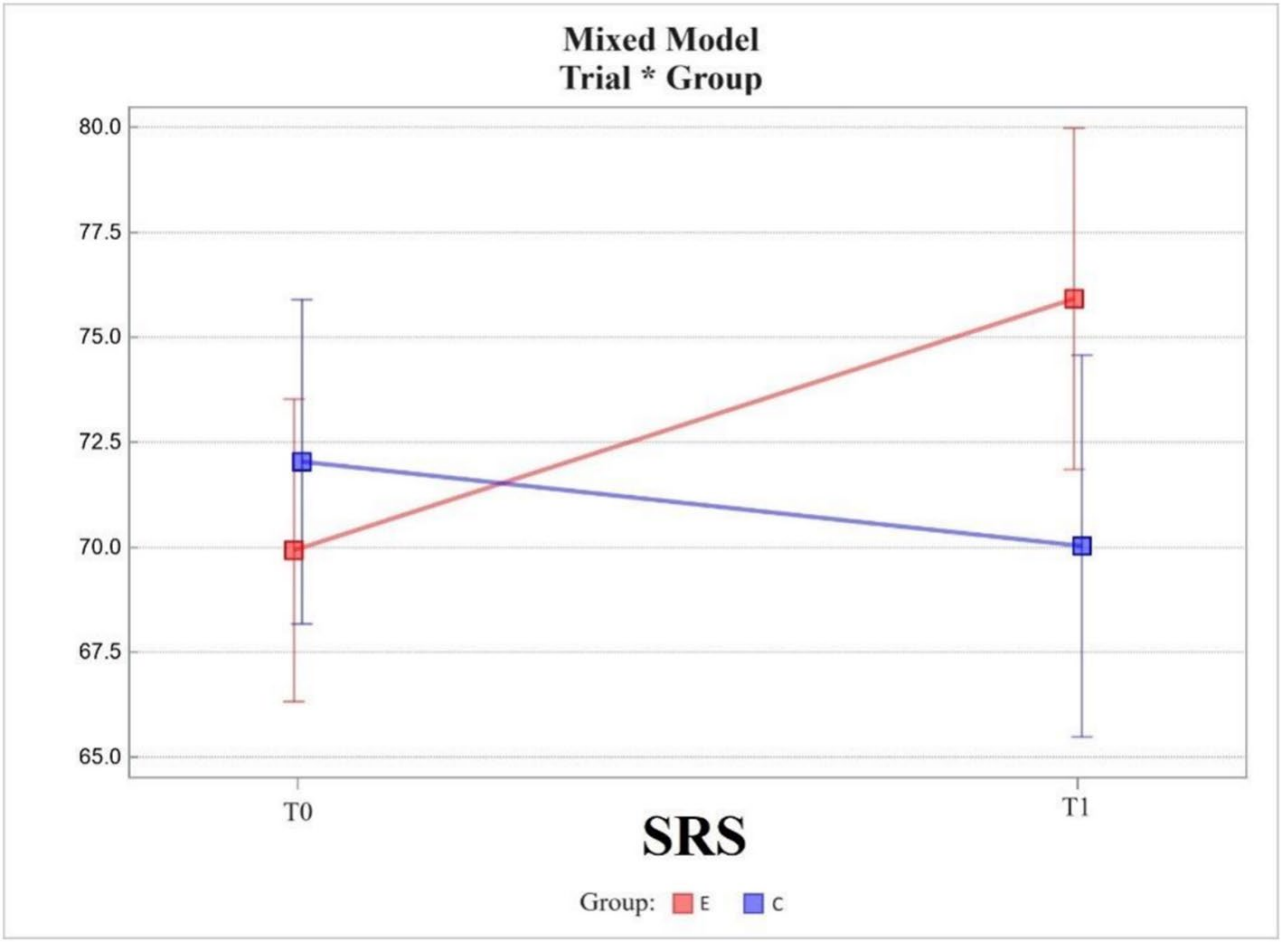
Mixed model – Stigma Resistance Scale (SRS); measurement points T0, T1

The change in scores between the T0 and T1 measurements in the experimental group differed from that in the control group, the difference between the two being 8.00 ± 2.50, which was statistically significant, *p* was 0.0020 (Fig. 5).

**Fig. 6:**
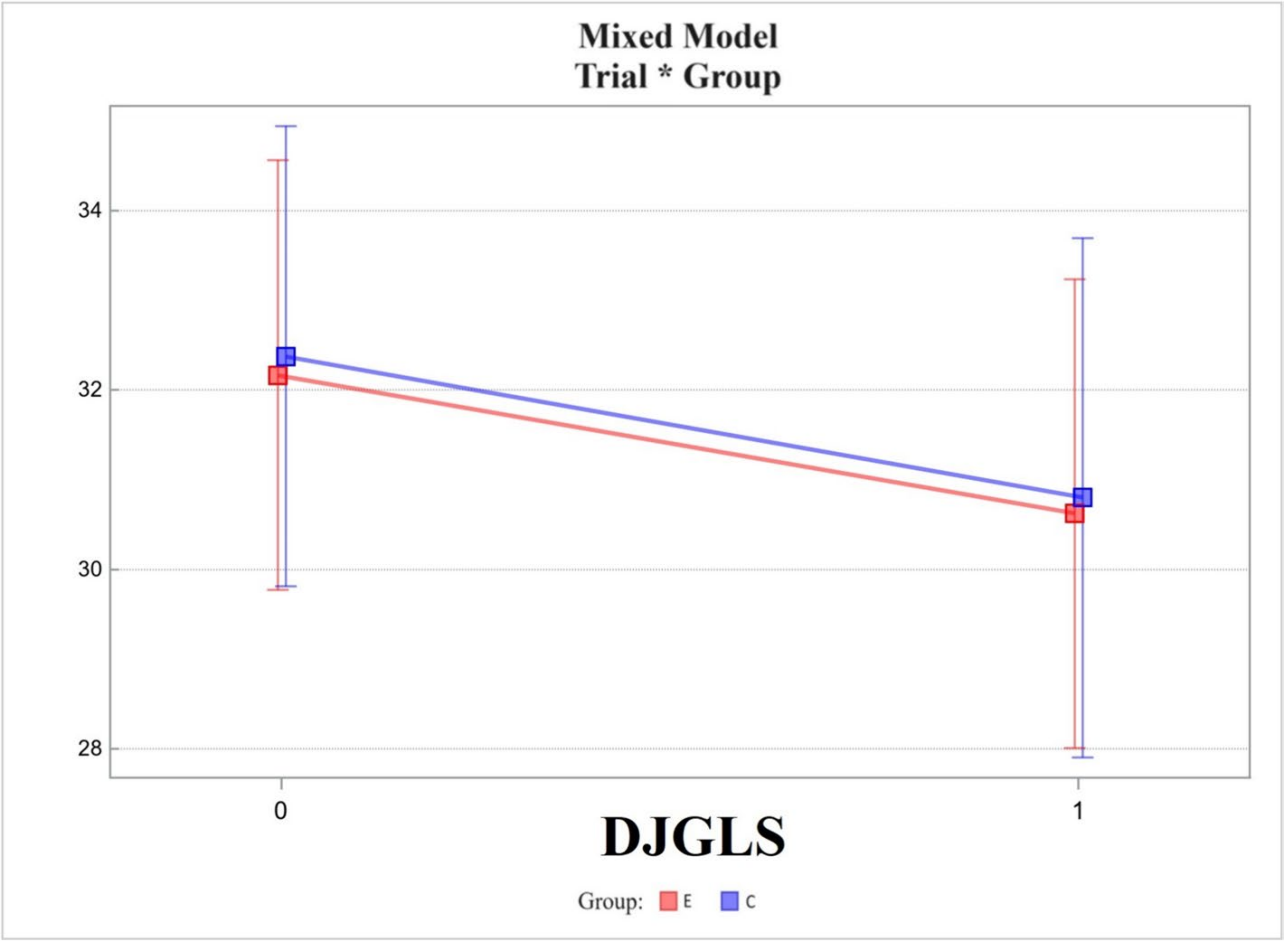
Mixed model – De Jong Gierveld Loneliness Scale (DJGLS); measurement points T0, T1

As for other measurement scales:

De Jong Gierveld Loneliness Scale (DJGLS):

- Changes in the experimental group did not differ from those in the control group, the difference between the two being -0.03 ± 1.41. The *p*-value was 0.981 (Fig. 6).

**Fig. 7:**
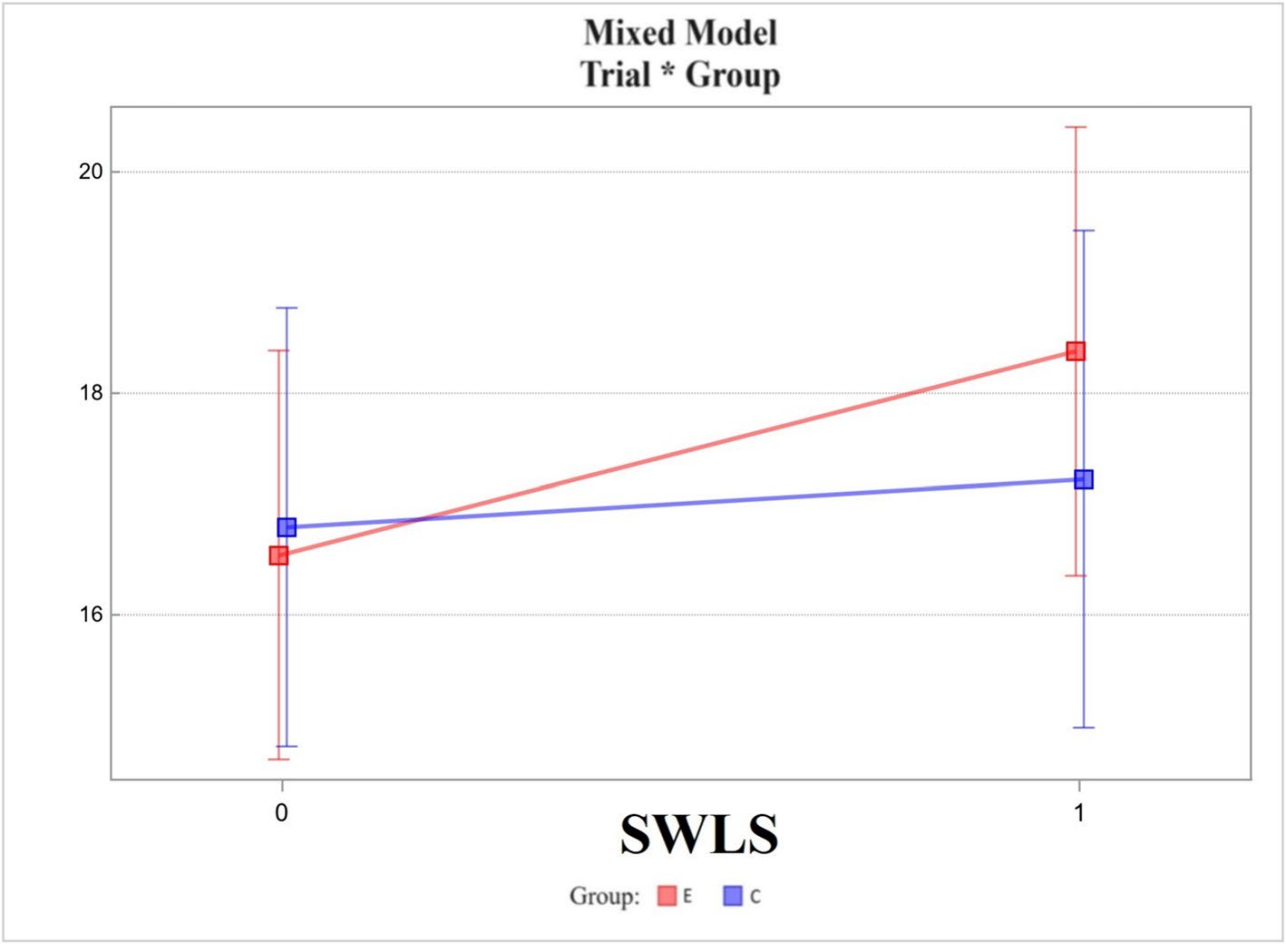
Mixed model – Satisfaction with Life Scale (SWLS); measurement points T0, T1

Satisfaction with Life Scale (SWLS):

- Changes in the experimental group did not differ from those in the control group, the difference between the two being 1.40 ± 1.11. The *p*-value was 0.212 (Fig. 7).

**Fig. 8:**
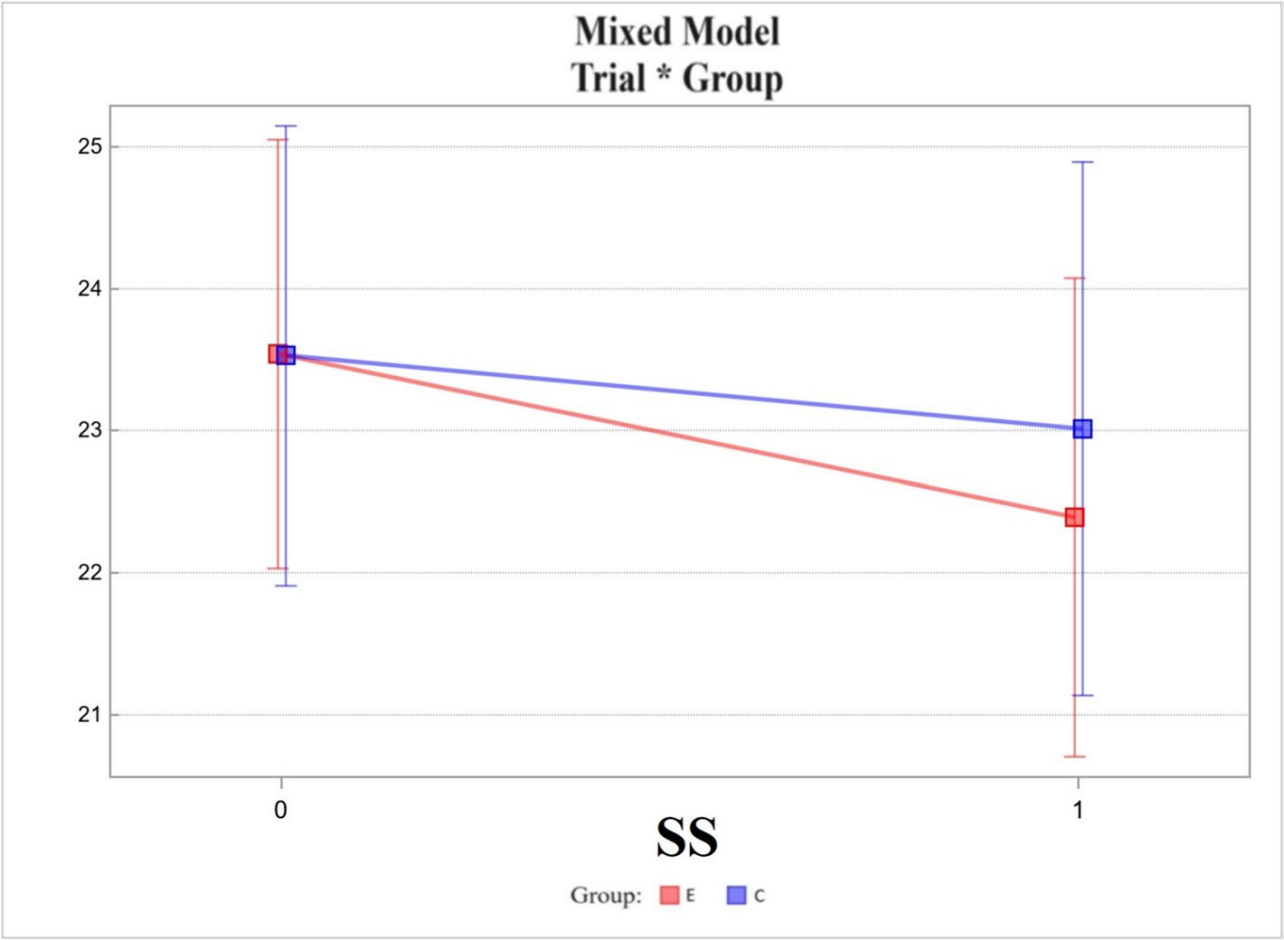
Mixed model – Secrecy Scale (SS); measurement points T0, T1

Secrecy Scale (SS) (Fig. 8):

- Changes in the experimental group differed from those in the control group, the difference between the two was 0.64 ± 1.00 and not statistically significant. The *p*-value was 0.527 (Fig. 8).

**Fig. 9:**
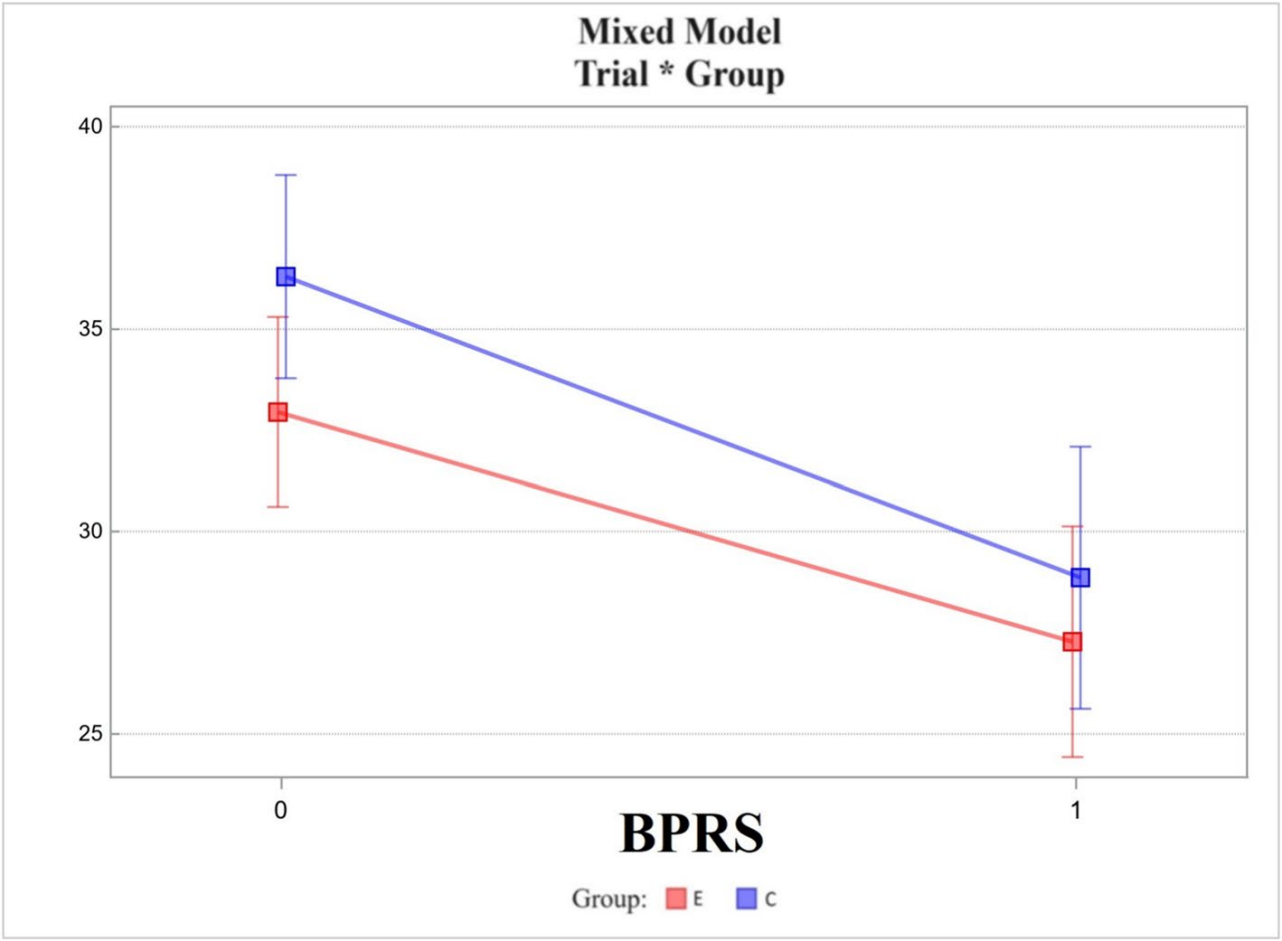
Mixed model – Brief Psychiatric Rating Scale (BPRS); measurement points T0, T1

Brief Psychiatric Rating Scale (BPRS):

- Changes in the experimental group did not differ from those in the control group, the difference between the two being -1.75 ± 2.16. The *p*-value was 0.420 (Fig. 9).

## Discussion

The described study focused on evaluating the effectiveness of a short-term CBT-based anti self-stigma intervention conducted in a psychiatric ward. To the best of our knowledge, the trial conducted was the first randomised trial of an intervention against self-stigma with a blinded evaluation of effectiveness.

A total of 104 patients were enrolled in the trial, of whom 27 patients dropped out of the trial before completion, representing 26% of participants. This figure is in a range comparable to other trials using cognitive behavioural therapy (average drop-out rate before therapy – 15.9%, during therapy – 26.2%) [62]. The reasons for not completing the therapy were most often related to the end of the hospitalisation, often a discharge at the patient’s request or a reluctance to extend the stay on the ward to complete the self-stigma intervention. The question concerning the reasons for not completing the therapy and thus the trial is complex and would require detailed analysis. We consider reasons such as a lack of motivation to continue the therapy, a lack of confidence in its effectiveness, or the difficulties patients faced in having to stay in a closed ward (the timing of the trial was during the COVID-19 restrictions).

The control group, as well as the experimental group, were derived from patients residing in an inpatient psychiatric ward, where patients stayed after psychiatric crises. Both groups were subjected to intensive therapeutic individual and group interventions, the aim of the interventions was to strengthen the recovery process and prevent further episodes of psychiatric crises. In addition to these interventions, the experimental group had the opportunity to benefit from an intervention to tackle self-stigma (“I am what I am”). Statistical analysis and comparison of the two trial groups at T0 indicated that the groups differed statistically significantly in terms of place of residence. The difference in the socio-demographic factor of place of residence may influence the results of the trial, but the analyses carried out did not find a correlation that would unambiguously indicate a relationship between this factor and the results of the scales. Individuals were allocated to groups through a randomisation mechanism, and we consider the result to be an “unanticipated” and “unplanned” effect of randomisation. Before the trial, we did not indicate that the groups were identical in terms of demographic variables [63, 64]. Apart from the residence parameter, there were no statistically significant differences between the two groups – experimental and control – at the T0 measurement point.

The primary endpoint of the trial was to assess the effectiveness of the self-stigma intervention in terms of the severity of internalised stigma as measured by the ISMI scale. The level of internalised stigma in both groups ranged in average values between 2.2 and 2.4, indicating a mild severity. On the ISMI scale, the changes in the control and experimental groups were statistically significant, considering the scores at the beginning and end of the trial; while in the experimental group, the change at T1 was not statistically significant compared to the control group. However, a statistically significant change was observed in both groups in the form of a reduction in the severity of internalised stigma. Previous research on the impact of therapeutic interventions “not targeted at self-stigma”, generally referred to as “rehabilitative or therapeutic interventions”, indicated a reduction in internalised stigma in the ISMI total score and a reduction in the number of people experiencing internalised stigma at > 2.5 [65–68]. The results presented here indicate that the conditions under which the trial was conducted (inpatient psychiatric ward, low baseline intensity of internalised stigma) may have influenced the ISMI scale scores.

Important changes observed in the trial were related to the secondary endpoints of the trial, including improvements in the Sense of Coherence (SOC-29) Questionnaire and the Stigma Resistance Scale (SRS). The results obtained seem interesting and indicate that it is worthwhile to refer to them in assessing the effectiveness of the therapy.

The salutogenic model of A. Antonovsky proposes a change in the approach to illness; it opposes seeing it in terms of its opposition to health and moves away from dichotomous thinking about health and illness. It focuses on the person’s life story and personal situation, not on the health problems and diagnosis. In this approach, individuals are seen as actively engaged in the search for health and self-fulfilment. The literature raises the topic of therapeutic interventions incorporating salutogenic approaches, and mixed interventions that do not belong to a single therapeutic paradigm are often described. Their common link is a focus on the salutogenesis premise, including comprehensibility, resources, improving coping strategies, and increasing personal potential [69, 70].

Stigma resistance is linked to the healing process. Questioning stigma and rejecting it is about the personal perspective. The rejection of stigma is a protective factor for the patient; it allows them to create an image of themselves that is not limited only to the disease aspects. In Livingston and Boyd’s study [22], a reduction in self-stigma and an increase in stigma resistance were related to recovery [36, 54, 71].

The intervention proposed to counteract self-stigma has several characteristic features describing it. It is part of a therapy whose focus stems from the principles of cognitive behavioural therapy. The working method included cognitive (discussion with automatic thoughts, beliefs and Socratic questioning and behavioural [behaviour modification]) techniques. The meetings were held in a group format and were conducted in a directive manner, i.e., each participant in the group had an active role in the meeting. The intervention has been planned for 10 meetings, taking 5 weeks to complete. This falls within the wide range of durations of interventions already used, from the well-researched for effectiveness NECT programme [44], which has a duration of 6 months, to short-term interventions developed for self-therapy [72]. The “I am what I am” intervention was conducted based on the protocol. The trial described here involved a relatively large group (considering the existing trials on the effectiveness of psychotherapy). Other interventions which were analysed, including the one described in Wood’s study [73], involved 30 people. NECT [40] is a programme with 119 participants in the trial.

There is limited research evaluating anti-stigma interventions conducted in psychiatric wards. The present study highlighted areas that should be considered in future similar research. Patients residing in therapeutic communities and undergoing intensive psychotherapy usually experience a reduction in self-stigma even when they are not subjected to any specific intervention. This means that to obtain statistically significant results, research needs to be conducted on a very large sample. This is not always feasible in ward conditions and often requires the involvement of many individuals. In our study, we did not analyse the long-term effects of therapy, which was suggested for future research. It would be particularly important to examine whether the improvement in recovery-related outcomes persists after discharge from the hospital. What we found to be a very valuable discovery was the emphasis on working with patients' personal resources and strengths after a mental health crisis, particularly in those hospitalised.

The results of the trial should be analysed in the light of its limitations. Its main limitation is the lack of assessment of the sustainability of the effects of therapy (follow-up). The trial was conducted under special conditions that affected the selection of patients into groups and the trial itself. Of these factors, the presence of intensive group and individual interventions, limited time of hospitalisation, and patients pre-qualified for the ward based on admission criteria appear to be important. Limitations related to the interpretation of the results of the trial include differences in demographic and residence-related data. Additional in-depth analysis would be required of the reasons for patients opting out of the intervention.

## Conclusions

In conclusion, the described intervention was well suited to the needs of the patients on the ward (as regards duration and conduct). The intervention “I am what I am” has proven to be effective especially in increasing the level of personal resources, primarily in increasing stigma resistance and sense of coherence, needed to build clinical and personal recovery and to counteract the negative consequences of self-stigma. The trial shows that when developing therapeutic protocols, in addition to targeting one aspect (internalised stigma), it is useful to simultaneously supplement the protocols with techniques that support patient resources.

## Data Availability

The data used in the article are available from the first author of the article, Katarzyna Frączek-Cendrowska, MA. e-mail: katarzfra@gmail.com

## Appendix

Table

**Table 4:** Description of therapy sessions, session duration: 60 min

Session No	Description
**1**	1. Introductions. Discussion of the aims of the group, the structure of the therapy and the principles of group work 2. Presentation of definitions of terms related to stigma: a) labelling b) stigma c) self-stigma 3. Demonstrating the negative consequences of stigma and self-stigma. The therapist tries to show the experiential dimension of the concepts discussed 4. Personal task
**2**	1. Discussion of the personal task 2. Imaginative exercise suggested by the therapist concerning the diagnosis 3. The therapist shows the link between self-stigma and low self-esteem and self-efficacy. Patients create their own models 4. Personal task
3	1. Discussion of the personal task 2. Discussion of methods to deal with stigma: (awareness of the stereotype, disagreeing with the stereotype, changing beliefs about stereotypes) 3. Introducing the principles of work based on the cognitive-behavioural model 4. Personal task
4	1. Discussion of the task 2. Psychoeducation on cognitive distortions 3. Personal task
5	1. Discussion of the personal task 2. Introducing a self-esteem assessment tool. Once completed, the therapist encourages patients to talk about the questionnaire and the results – reflections 3. The therapist analyses together with the patients whether self-stigma affects low self-esteem 4. Personal task
6	1. Discussion of the personal task 2. Introducing the concepts of core beliefs, “rules for living” 3. Introducing an exercise to find the core beliefs about oneself and the accompanying “rules for living” Personal task
7	1. Discussion of the personal task 2. The therapist demonstrates and discusses the factors that sustain low self-esteem 3. Introduction of the exercise: patients analyse the factors that influence their sense of worth 4. Personal task
8	1. Discussion of the personal task 2. Role-play exercise – discussion with a negative self-schema 3. Personal task
9	1. Discussion of the personal task 2. Analysis of own characteristics, finding personal resources 3. Personal task
10	1. Discussion of the personal task 2. Summary and conclusion of therapy

4
